# Hemodynamic changes in superficial arteriovenous malformation surgery measured by intraoperative ICG fluorescence videoangiography with FLOW 800 software

**DOI:** 10.1186/s41016-020-00208-y

**Published:** 2020-08-10

**Authors:** Xun Ye, Liang Wang, Ming-tao Li, Xiao-lin Chen, Hao Wang, Li Ma, Rong Wang, Yan Zhang, Yong Cao, Yuan-li Zhao, Dong Zhang, Shuo Wang

**Affiliations:** 1 Department of Neurosurgery, Beijing Tian Tan Hospital, Capital Medical University, Beijing, 100050 China; 2 Department of Neurosurgery, Tianjin Fifth Center Hospital, Tianjin, 300450 China; 3 Department of Neurosurgery, Peking University International Hospital, Beijing, 102206 China

**Keywords:** Arteriovenous malformations, FLOW 800 software, Hemodynamics, Indocyanine green

## Abstract

**Background:**

Arteriovenous malformation(AVM) have long-term “blood stealing” characteristics, which result in complicated hemodynamic features. To analyze the application of intraoperative indocyanine green angiography with FLOW 800 software in AVM surgeries.

**Methods:**

Data on 17 patients undergoing surgery with ICG fluorescence were collected in Beijing Tiantan Hospital. To analyze the hemodynamic features of AVM and the influence on the peripheral cortex of AVM resection, we assessed the following hemodynamic parameters: maximum intensity, slope of rise, time to half-maximal fluorescence, and transit time from arteries to veins.

**Results:**

In the 17 superficial AVMs studied, the time-delay color mode of the FLOW 800 software was superior to the traditional playback mode for identifying feeding arteries, draining veins, and their relation to normal cortical vessels. The maximum fluorescence intensity and slope of the ICG fluorescence curve of feeder arteries and draining veins were higher than those of normal peripheral vessels (*P* < 0.05). The transit times in AVMs were significantly shorter than those in normal peripheral vessels (*P* < 0.05). After AVM resection, cerebral flow increased in the cortex, and local cycle time becomes longer, although the differences were not significant (*P* > 0.05).

**Conclusions:**

Hemodynamic parameter analysis provided quality guidance for the resection of AVMs and could also be used in estimating changes in blood flow in the local cortex to identify abnormal hyperperfusion and residual nidus.

## Background

Arteriovenous malformations (AVMs) are responsible for about 2% of all hemorrhagic strokes, mainly in children and young adults [1, 2]. Despite advances in endovascular treatment and radiotherapy, surgical resection remains the most effective treatment for AVMs. Therefore, real-time vascular imaging and hemodynamic monitoring are necessary for surgical strategy adjustments during AVM surgery.

Intraoperative indocyanine green (ICG) videoangiography is used widely in neurosurgery and has proven a useful addition to neurovascular surgery [3–6]. FLOW 800 is a recently developed visualization, cinefluorography, and data analysis software that can be installed in the Carl Zeiss OPMI Pentero or OPMI Pentero 900 surgical microscopy system to analyze hemodynamic data. The time-delay color map provided by the software employs colors to instantly identify the direction and sequence of blood flow. The intensity diagram function helps to visualize variations in blood flow over time.

In this study, we analyzed hemodynamic changes in AVM nidus and normal peripheral vessels during surgery using ICG videoangiography and the FLOW 800 software.

### Ethics

The study protocol and written informed consent form were reviewed and approved by the Institutional Review Board of Beijing Tiantan Hospital (Beijing, China), which is affiliated with Capital Medical University (Beijing, China) (KY 2017-012-02).

## Methods

### Patients

We enrolled 17 patients who were admitted to our department with AVM during March to November 2016 (Table 1) in Beijing Tiantan Hospital. All patients underwent AVM resection surgery under general anesthesia.

**Table 1:** Summary of the AVM patients

Patient	Age	Sex	Presentation	S-M grade	Location	Size (cm)	No. of cortex ROI		No. of nidus ROI		No. of ICG videos	Postoperative DSA/CTA	MRS
							A	V	F	D			
1	55	F	Headache	4	L,PT	5.0 × 4.0 × 5.0	3	2	2	3	3	No	4
2	24	M	Hemorrhage	3	R,P	4.0 × 3.0 × 3.0	2	2	3	2	3	No	3
3	24	M	Hemorrhage	3	L,P	4.0 × 3.0 × 3.5	3	3	2	2	3	CTA	4
4	28	M	Headache	2	R,FT	4.5 × 3.5 × 3.0	4	3	2	2	3	CTA	0
5	40	M	Hemorrhage	3	R,O	5.5 × 4.0 × 2.5	3	3	2	4	3	DSA	1
6	33	M	Epilepsy	2	L,P	3.0 × 3.5 × 4.0	2	2	1	2	3	NO	0
7	44	M	Epilepsy	2	R,F	3.5 × 2.5 × 2.5	3	4	4	2	3	DSA	0
8	30	M	SAH	3	L,T	5.0 × 3.5 × 3.0	2	2	2	1	2	CTA	0
9	31	M	Epilepsy	4	R,F	4.5 × 4.5 × 4.0	4	3	3	2	3	DSA	0
10	45	F	Hemorrhage	3	L,TO	4.5 × 4.5 × 4.0	3	3	4	4	3	DSA	2
11	26	M	Hemorrhage	2	L,P	4.0 × 4.0 × 4.0	2	2	3	2	3	DSA	3
12	18	F	Epilepsy	3	R,F	4.5 × 4.0 × 4.0	3	2	2	2	4	DSA	0
13	13	M	Headache	3	R,F	4.0 × 4.5 × 3.0	2	2	3	2	3	DSA	0
14	28	M	Headache	2	L,T	3.5 × 3.0 × 2.5	2	3	2	3	3	DSA	0
15	38	F	Epilepsy	4	R,T	6.0 × 4.5 × 4.0	3	3	3	4	3	CTA	1
16	29	F	Epilepsy	3	L,F	3.5 × 3.0 × 4.0	3	2	4	4	3	CTA	0
17	34	M	Headache	2	R.O	4.5 × 4.0 × 4.0	2	2	2	3	3	DSA	0

### ICG

ICG is a nontoxic tricarbocyanine dye (molecular weight 775 Da, C43H47N2O6 S2Na) with peak spectral absorption at 805 nm and peak emission at 835 nm [7]. In each patient, ICG dye was injected into a peripheral vein as a bolus (standard 25-mg dose dissolved in 5 mL water or 0.25 mg/kg). Fluorescence angiography was typically performed at three points during the AVM resection. First, ICG videoangiography was performed to obtain information about the hemodynamics of the AVM and normal cortical vessels surrounding the AVM before the resection. This step was a primary, superficial survey. Second, ICG videoangiography was repeated to obtain information on hemodynamic changes in the AVM after the major feeders were clipped. This step served to confirm the effect of clipping and to gather data for further hemodynamic analysis. Finally, ICG analysis was performed at the end of the resection to identify any residual AVM and mark the cortical vessels for further analysis.

### Operation

All operations were performed using two microscopes available in our department (Carl Zeiss OPMI Pentero microscopes with INFRARED 800 camera modules; Carl Zeiss, Oberkochen, Germany). Both microscopes had near-infrared video integration and were able to perform ICG videoangiography.

Under illumination with a near-infrared light source (excitation range 700–780 nm), real-time microscope images were recorded. An optical filter that allowed only fluorescence in the ICG emission range (820–900 nm) was used. To obtain optimum illumination, we set the standards for AVM resection as follows: the microscope diaphragm was automatically adjusted to a wide setting; the illuminated field diameter was set in the middle position; the illumination intensity was set to 50%; the recommended working distance of the scope was set at 300 mm; and the Pentero microscope zoom was set to 5 ×.

ICG fluorescence angiography clearly showed the AVM nidus. Feeder arteries and draining veins were mainly chosen as the hotspots. We selected three to five feeder arteries and draining veins, along with peripheral cortical arteries, as the hemodynamic parameter collection points using the FLOW 800 software.

### FLOW 800 software and data analysis

FLOW 800 is an analytic color visualization tool that can evaluate the fluorescence video sequences obtained by microscope-integrated ICG fluorescence angiography using INFRARED 800. It provides an objective evaluation of results, visually and in different colors. Using this visualization tool, the ICG transit curve intensities were recorded, and maps of maximal fluorescence intensities were saved. Then, two-dimensional visual maps (including color maps) of the AVM were created according to maximal fluorescence intensities or time to half-maximal fluorescence. The intensity diagram function can provide a variable shape that reflects variation in the blood flow of the feeders, drainers, and peripheral vessels before and after AVM resection. Related parameters were defined as follows: time to half-maximal fluorescence was defined as the time required for the ICG fluorescence intensity to reach 50% of the maximum value (T1/2 peak); transit time was defined as the time required for blood to flow from artery to vein (arteriovenous transit time); rise time was defined as the interval between 10% and 90% of the maximum signal; and cerebral blood flow index slope was defined as the ratio of maximum fluorescence intensity to rise time.

### Qualitative ICG video analysis

The indication for each ICG video during the AVM resection was recorded. As recommended by Hanggi et al. [8, 9], the ICG videos were classified into three categories based on the phase of AVM surgery: (i) primary resection (pre-resection), (ii) intra-resection, and (iii) post-resection. Positive and negative attributes of each ICG video recording were recorded. In addition, the suitability of the ICG video for flow analysis was noted. If the video was suboptimal for fluorescence intensity flow analysis, the reason was clearly recorded. The ICG videos from the 17 patients were retrospectively reviewed by an independent third-party neurosurgeon and were compared to the concurrent normal white-light video recording.

### Statistical analysis

Values are presented as the mean ± standard deviation. The statistical analysis was performed using the Statistical Package for the Social Sciences ver. 16.0 (SPSS, Chicago, IL, USA). The mean measurements of the computed tomographic angiography (CTA) and three-dimensional model were compared using one-way analysis of variance followed by Fisher’s least significant difference test. Comparisons between two groups (survey scores) were achieved using Student’s *t* test. Two-tailed *P* values < 0.05 were considered statistically significant.

## Results

### Patient outcomes

Our study enrolled 17 AVM patients who were judged suitable for surgical resection surgery under general anesthesia. The demographic characteristics of the patient population are listed in Table 1. Equal numbers of male and female patients were enrolled, and the mean age was 34.5 years (range 18–55 years). Eight patients had grade 3 Spetzler–Martin AVM, six had grade 2 AVM, and 3 had grade 4 AVM. The clinical presentation was hemorrhage in 35.3% of patients, including one subarachnoid hemorrhage, epilepsy in 35.3%, and headache in 29.4% of patients.

A total of 51 ICG fluorescence angiographies were performed; 15 patients received angiography three times, 1 received it two, and 1 patient received angiography four times. Blood pressure was maintained at 100–120 mmHg (105 ± 12 mmHg). Fourteen patients underwent digital subtraction angiography (DSA) or CTA after the operation, and all tests showed that the AVM was totally removed.

No side effects or adverse reactions were noted after ICG administration in any of the patients.

### Quantitative ICG fluorescence intensity analysis

**Fig. 1:**
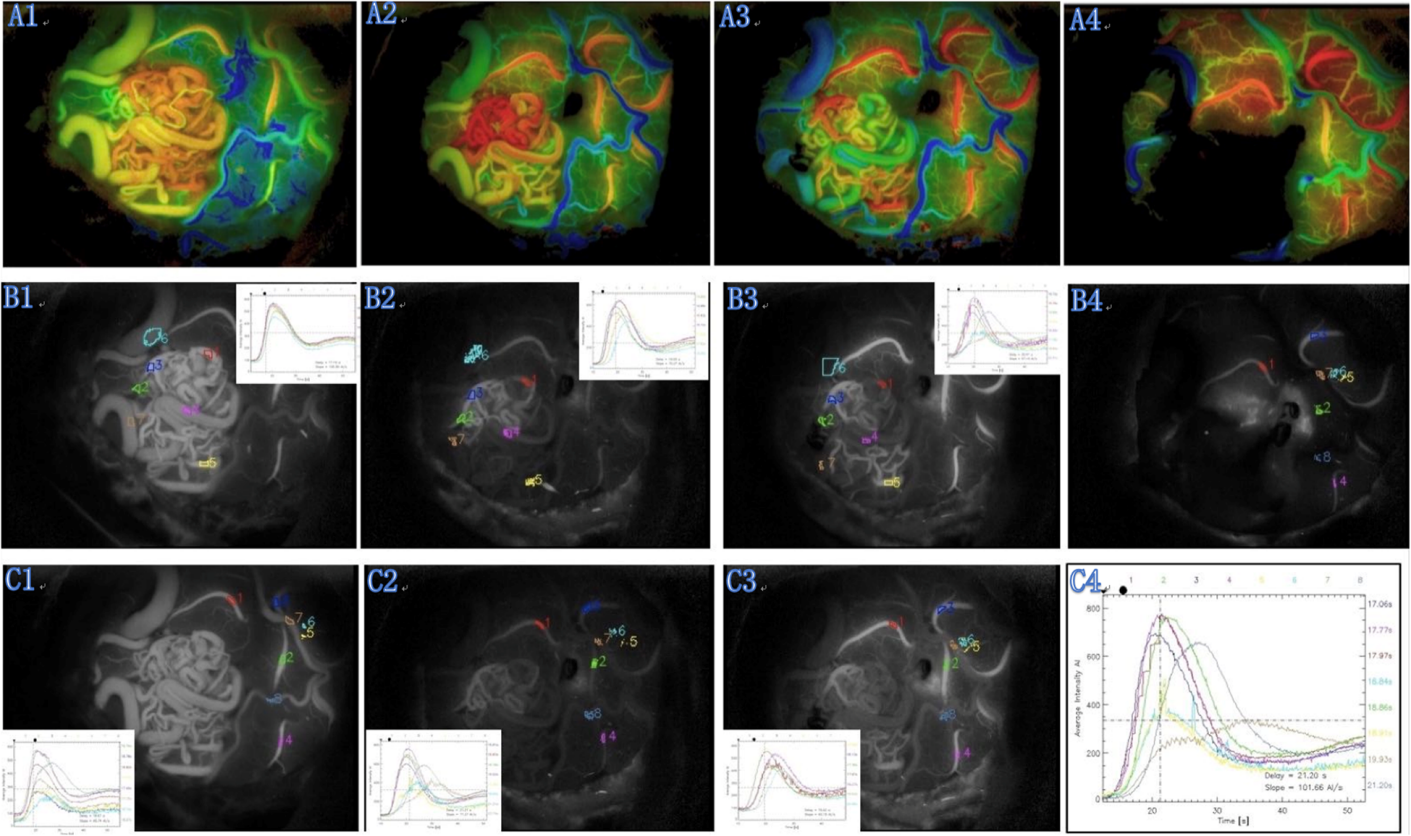
The typical ICG fluorescence intensity analysis of AVM before and after surgery. **A1** The AVM hemodynamics characteristics before clipping feeding artery. **B1** The typical picture of ICG video, hemodynamic parameter acquisition area of the nidus before resection, and the fluorescence intensity curve analysis chart. **C1** The typical picture of ICG video, hemodynamic parameter acquisition area of the cortex vessels before resection, and the fluorescence intensity curve analysis chart. **A2** The AVM hemodynamics characteristics after clipped one feeding artery. The blood increased in part of AVM. **B2** The typical picture of ICG video, hemodynamic parameter acquisition area of the nidus after clipping one feeding artery, and the fluorescence intensity curve analysis chart. **C2** The typical picture of ICG video , hemodynamic parameter acquisition area of the cortex vessels after clipping one feeding artery, and the fluorescence intensity curve analysis chart. **A3** The AVM hemodynamics characteristics after clipped two feeding artery. Some communicating artery open. **B3** The typical picture of ICG video, hemodynamic parameter acquisition area of the nidus after clipping two feeding artery, and the fluorescence intensity curve analysis chart. **C3** The typical picture of ICG video , hemodynamic parameter acquisition area of the cortex vessels after clipping two feeding artery, and the fluorescence intensity curve analysis chart. **A4** After resection of AVM, cortex vessel blood increased. **B4** The typical picture of ICG video and hemodynamic parameter acquisition area of the cortex vessels after surgery. **C4** The fluorescence intensity curve analysis chart of cortex vessels after surgery

**Fig. 2:**
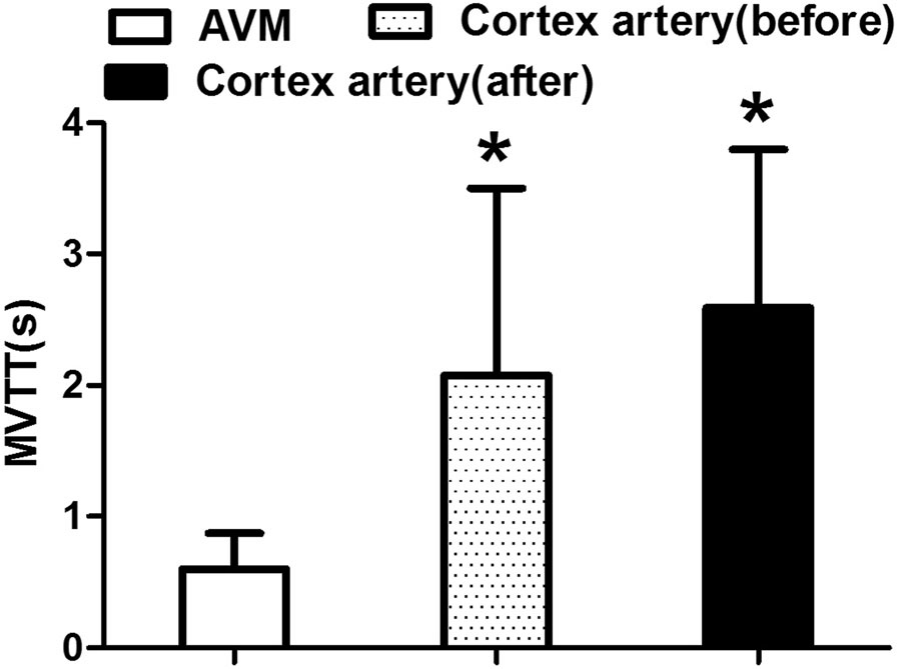
MVTT of AVM, cortex artery before and after AVM surgery. (*P < 0.05 compare to the AVM) MVTT microvascular transit time

In the 17 superficial AVMs, the time-delay color mode provided by the FLOW 800 software proved more useful than the traditional playback mode in identifying feeding arteries, draining veins, and their relations to normal cortical vessels. The transit times in AVMs were significantly shorter than those in normal peripheral vessels (*P* < 0.05; Figs. 1 and 2).

### Arterial feeders

**Fig. 3:**
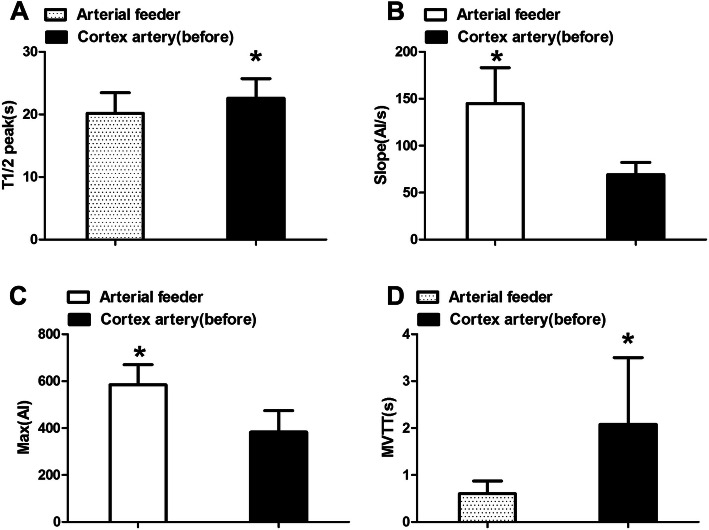
Hemodynamic changes of feeding artery compare to the cortex artery before surgery (*P < 0.05 compare to the other group). T1/2 peak, time to half-maximal fluorescence; Slope, slope of rise; Max, maximum fluorescence intensity; MVTT, microvascular transit time

The development time of feeder arteries (T1/2 peak 20.19 ± 3.16 s) was significantly earlier than that for cortical arteries (T1/2 peak 22.59 ± 3.13 s), which could serve as a basis for identifying feeder arteries. The slope of arterial feeders (144.95 ± 38.08 AI/s) was significantly greater than that of normal cortical arteries (69.20 ± 13.05 AI/s; *P* < 0.05), indicating that AVMs exhibited hemodynamic characteristics of high flow and high perfusion (Fig. 3).

### Draining veins

**Fig. 4:**
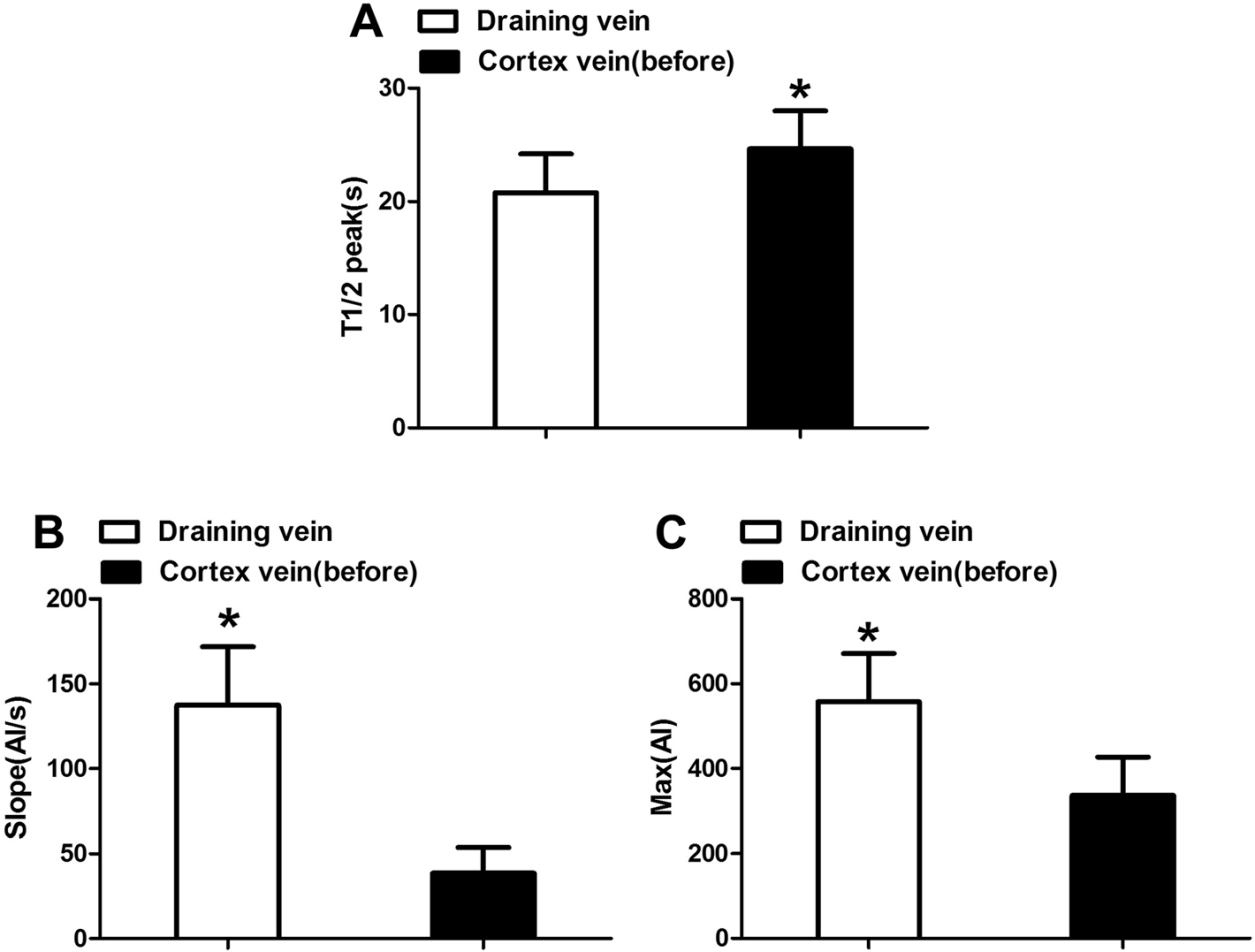
Hemodynamic changes of draining vein compare to the cortex vein before surgery (*P < 0.05 compare to the other group). T1/2 peak, time to half-maximal fluorescence; Slope, slope of rise; Max, maximum fluorescence intensity

We analyzed the T1/2 peak, slope, and maximum fluorescence intensity of draining veins; The maximum fluorescence intensity (336.47 ± 90.41), slope of the ICG fluorescence curve (178.66 ± 15.09), and T1/2 peaks (20.79 ± 3.42 s) of draining veins were higher than those of normal peripheral vessels (*P* < 0.05). This observation could be helpful in identifying draining veins (Fig. 4).

### Cortical arteries

**Fig. 5:**
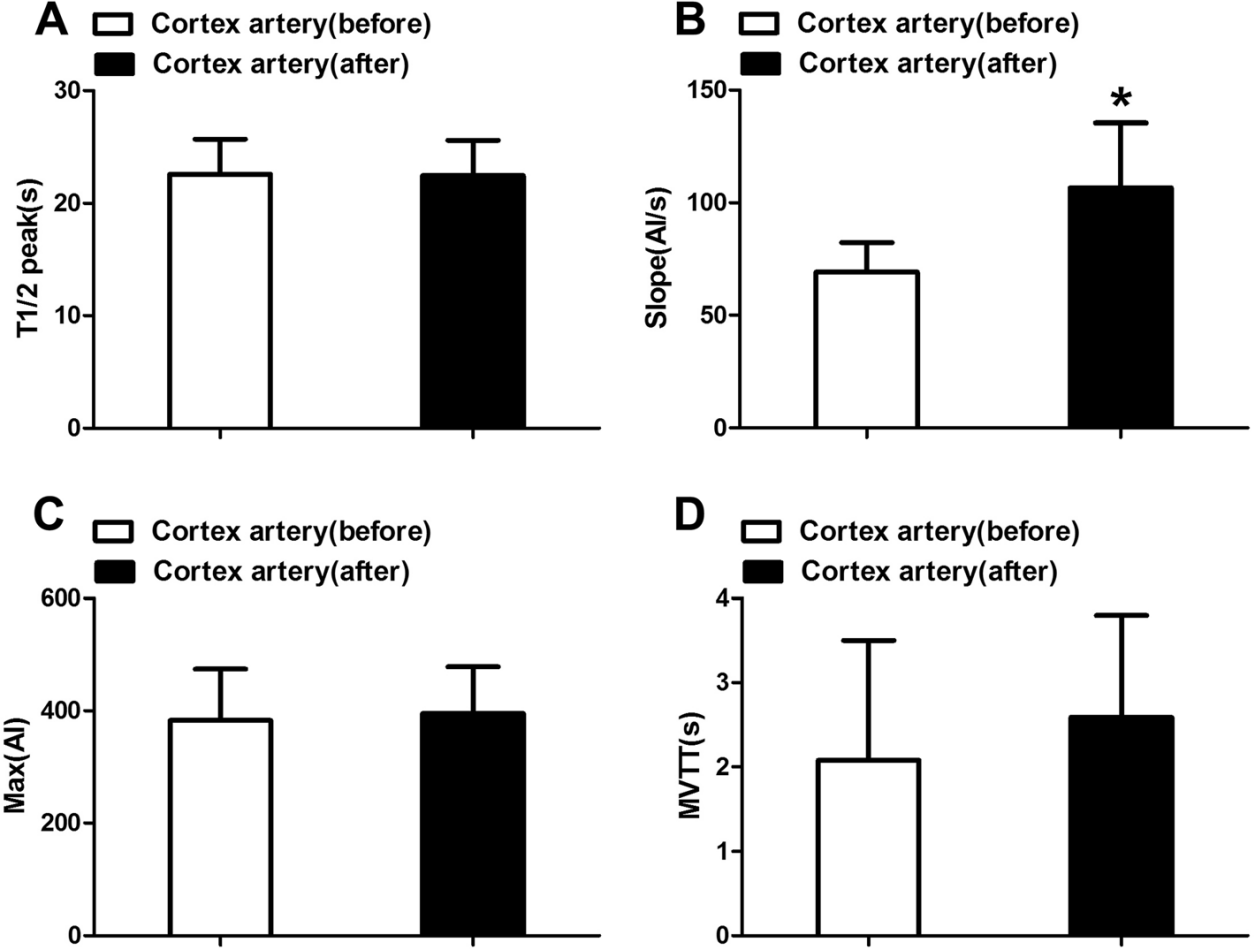
Hemodynamic changes of cortex artery before and after surgery (*P < 0.05 compare to the other group). T1/2 peak, time to half-maximal fluorescence; Slope, slope of rise; Max, maximum fluorescence intensity; MVTT, microvascular transit time

Before AVM resection, the T1/2 peak of the cortical arteries was 22.59 ± 3.13, and the slope was 69.20 ± 13.05. After AVM resection, the T1/2 peak was 22.45 ± 3.15 s, and the slope was 106.63 ± 28.85 (*P* < 0.05). The microvascular transit time (MVTT) of the cortical arteries was 2.08 ± 1.42 s before and 2.59 ± 1.21 s after resection. The cerebral flow in the cortex increased, and the local cycle time became longer, although these differences were not significant (*P* > 0.05; Fig. 5). Furthermore, the results showed that cortical blood perfusion was increased, with some local areas exhibiting hyperperfusion. The delayed cycling time showed that AVM resection may have caused local cortical venous reflux (Fig. 5).

### Cortical veins

**Fig. 6:**
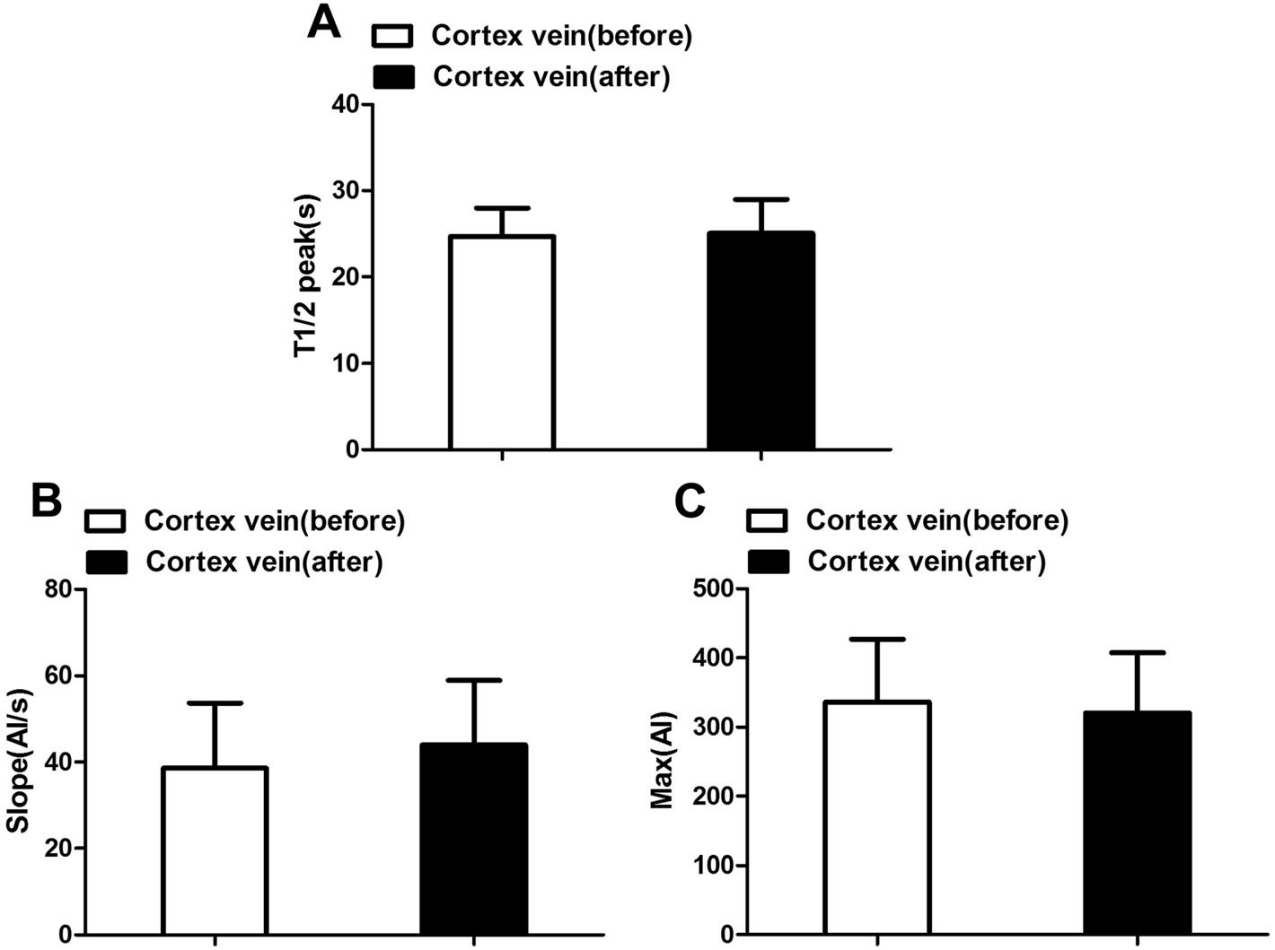
Hemodynamic changes of cortex vein before and after surgery. T1/2 peak, time to half-maximal fluorescence; Slope, slope of rise; Max, maximum fluorescence intensity

Before AVM resection, the T1/2 peak of the cortical veins was 24.67 ± 3.31 s, and the slope was 137.44 ± 34.42. After resection, the T1/2 peak was 25.04 ± 3.31 s, and the slope was 38.59 ± 15.09; the MVTT of cortical veins was 2.08 ± 1.42 s before and 2.59 ± 1.21 s after resection (Fig. 6).

Although we found that cerebral flow in the cortex increased and local cycle time became longer, these differences were not significant (*P* > 0.05). Nonetheless, the results showed the tendency of AVM removal to affect the cortical veins.

## Discussion

Microscope-integrated near-infrared ICG videoangiography has been used regularly in vascular neurosurgery for several years. FLOW 800 is a reliable and useful addition to microscope-integrated color ICG videoangiography [9]. The color map can easily identify the feeder arteries, draining veins, passage arteries, cortical arteries, and cortical veins. Time–intensity curves for regions of interest (ROIs) facilitate semiquantitative hemodynamic analysis and real-time monitoring of AVMs and cortical vessels. In this study, we employed a simple method to measure the hemodynamic parameters of AVMs and cortical arteries using the ROI time–intensity curves provided by FLOW 800. The results showed that the maximum fluorescence intensity and the slope of the ICG fluorescence curve of feeder arteries and draining veins were higher than those of normal peripheral vessels. The transit times in AVMs were significantly shorter than those in normal peripheral vessels.

AVMs have long-term “blood stealing” characteristics, which result in decreased perfusion pressure in brain tissue around AVM lesions, reduced cerebral vascular resistance and automatic regulation capacity of vascular. Miyasaka et al. [10] showed that the feeding arterial pressure of AVM is lower than that of normal cortical arteries, and the intravenous drainage pressure is significantly higher than that of normal veins. The results of our AVM hemodynamic parameter analysis revealed hemodynamics characterized by high blood flow and low resistance. The developing time of feeder arteries was significantly earlier compared to that of cortical arteries, the transit times in AVMs were significantly shorter than those in normal peripheral vessels, and the slope of the time–intensity curve for cortical arteries increased significantly following surgical resection of AVM.

An analysis of the blood flow parameters of peripheral cortical arteries before resection showed that the cortical perfusion of AVMs was lower than that in normal tissues, suggesting a relative lack of blood supply to the cortex; additionally, the density of the cortical layer around the vascular malformation was greater than that of normal cortical blood vessels, and cortical capillaries proliferated.

In a previously published hemodynamic study, Ng et al. showed that ICG videoangiography was useful in different phases of AVM surgery [11]. Furthermore, the results of further semi-quantitative flow analysis using FLOW 800 software showed it to be useful in confirming the lack of evidence of nidus in the exposed resection cavity and the absence of flow in the main draining vein. Ng et al. [11] measured the average T1/2 peak intensities and arteriovenous T1/2 peak time, as well as the T1/2 peak fluorescence rates for the arterial feeder, draining vein, and normal cortex. The results of our AVM study of the nidus were congruent with those of Ng et al. [11]. Our study further focused on flow changes in the cortical artery and vein. We found that the cerebral flow in the cortex increased and local cycle times become longer, although the differences were not significant (*P* > 0.05). The results showed that cortical blood perfusion was increased; in some local areas, hyperperfusion was noted. The delayed cycling time showed that AVM resection may have caused local cortical venous reflux.

After AVM resection, the slope of ICG fluorescence in the peripheral cortical arteries was significantly increased (from 69.20 to 106.63), the maximum fluorescence intensity was enhanced, and image develop of cortical arteries was earlier than before surgery. This showed that cortical perfusion was increased after the AVM resection; indeed, some parts exhibited hyperperfusion. However, the time of circulation was prolonged, suggesting venous reflux due to obstruction in some parts of the cortex, a phenomenon termed “venous overload.” Wilson et al. proposed that venous overload was caused by thrombosis in a draining vein due to clogging by an AVM remnant [12]. Our results also supported the hypothesis that severe cortical hyperperfusion and venous overload could induce normal perfusion pressure breakthrough, the main source of postoperative hemorrhage and cerebral swelling in AVM. The slope of the time–intensity curve for cortical arteries increased significantly following the surgical resection of AVM, reflecting an increase in cerebral flow in peripheral cortical regions after AVM resection.

We compared the T1/2 peak fluorescence rate, slope, and maximum fluorescence intensity of the draining and cortical veins. The results showed that the timing of the T1/2 peak fluorescence rates was earlier for the draining than for the cortical veins, and the slope and maximum fluorescence intensity of the draining veins were greater than those of the cortical veins. These findings may prove useful in identifying abnormal draining veins. The real-time feedback regarding blood flow provided by FLOW 800 could help the surgeon to determine or modify the operative strategy for a safe and complete AVM resection [11].

### Comparison and limitations

A combination of intra- and postoperative DSA is regarded as the gold standard for identifying AVM remnants after surgery, especially in small and deep AVM surgery [13]. But disadvantages are also apparent. In addition to the time consumed and the radioactive risk, DSA is invasive, and the complication rate is 0–4.2% [14, 15]. Fluorescent angiography with intraoperative visualization of cerebral arteries has achieved widespread use owing to its greater ease of use, rapid delivery, and patient safety compared with DSA. However, this technology also has its limitations. ICG videoangiography can only observe the blood vessels under microscope, and it requires sufficient direct exposure of the surgical field without any shield. Arterial wall calcification or AVM presenting with hemorrhage could affect visibility. Furthermore, for deep AVMs, the microscope light is difficult to insert, and ICG image quality is likely to suffer accordingly [16–20]. By contrast, intraoperative ultrasound is another convenient and faster tool that has the advantage of confirming the location of deep AVMs, but it is difficult to distinguish between feeding and draining arteries using ultrasound [21].

## Conclusion

Intraoperative ICG videoangiography combined with analysis of hemodynamic parameters obtained by the FLOW 800 software is a convenient and effective method for evaluating the hemodynamic features of superficial AVMs by identifying the feeding arteries, draining veins, crossing arteries, and cortical vessels, and to provide quality guidance for AVM resection. Furthermore, hemodynamic parameter analysis can be used for estimating changes in blood flow in the local cortex, thereby identifying residual nidus and vessels contributing to abnormal hyperperfusion.

## Data Availability

Please contact author for data requests.

## Abbreviations

AVMs: Arteriovenous malformations
ICG: Intraoperative indocyanine green
CTA: Computed tomographic angiography
DSA: Digital subtraction angiography
MVTT: Microvascular transit time
